# The educational value of an audience response system use in an Iraqi medical school

**DOI:** 10.1186/s12909-022-03381-z

**Published:** 2022-04-26

**Authors:** Faiz Tuma, Husam Majeed, John Blebea, Aussama Nassar, William C. Durchholz, Susie Schofield

**Affiliations:** 1 Central Michigan University College of Medicine, PO Box 4181, Saginaw, MI 48606 USA; 2 Western Michigan University Homer Stryker MD School of Medicine, Kalamazoo, USA; 3 Wasit University College of Medicine, Kut, Iraq; 4 School of Medicine, Stanford University, Stanford, USA; 5 School of Medicine, University of Dundee, Dundee, United Kingdom

**Keywords:** Educational Technology, Education, Medical, Students, Medical, Iraq, Surveys and Questionnaires, Educational Measurement

## Abstract

**Background:**

Medical education is continually evolving particularly through the modern implementation of educational technology. Enhancing interactive learning in the classroom or lecture settings is one of the growing uses of educational technology. The role and potential benefits of such technology may not be as evident in developing educational systems like the one in Iraq. The purpose of this study was to examine the effect and perception of the use of an audience response system (ARS) on interactive medical education in Iraq. A mixed quantitative and qualitative research methodology approach was used to study the effects and users’ perceptions (both student and tutor) of the ARS.

**Method:**

The study was conducted in an Iraqi medical school in the Head and Neck course during the spring semester for third-year medical students. The course involved fifteen one-hour lectures over fifteen weeks. Users’ perceptions were evaluated by survey and focus group discussions (FGD). Descriptive statistics were used for quantitative measures and thematic analysis for the qualitative data.

An ARS system was installed and integrated into the course lectures throughout the course period of three months to enhance interactive learning. Three to five interactive questions were used in each lecture. Anonymous participation and answers were maintained. The appropriate discussion was initiated when pertinent depending on students’ answers.

**Result:**

Most students (77% of survey, 85% of FGD) perceived the use of ARS as impactful on their learning.

They found the ARS engaging (70%), motivating (76%), promoting interactions (73%), and augment learning through better understanding and remembering (81%). Through the FGD, students expressed improved focus, enhanced thinking and reflection, and joyful learning. The educator perceived the ARS use as practical, interactive, thinking-stimulator, and reflective of student’s understanding. The required technology skills were reasonable; however, it demanded extra non-insignificant time to learn the use.

**Conclusion:**

The perception of the ARS in this study was overall positive, providing encouragement for wide application of this technology in medical education in the developing world. Further studies are needed to validate and prioritize ARS usage in medical education in Iraq.

**Supplementary Information:**

The online version contains supplementary material available at 10.1186/s12909-022-03381-z.

## Introduction

Medical education is continually evolving with very significant changes having taken place during the last two decades [1]. There has been a gradual shift in medical trainees’ education in various aspects including the traditional lectures where delivery of information is the focus of the activity to a more engaging and participatory style of teaching. This is, in part, due to the increasing evidence that lectures alone (as a method of delivering information) are not effective in solidifying long-term knowledge acquisition nor in promoting its application to the clinical setting [2, 3]. There is an increasing trend toward self-directed learning that actively engages students in enquiry-based learning [4]. The utilization and extent of this trend in developing educational systems such as Iraq are not well known.

Another change has been the utilization of educational technology on medical curricula. This has been partly driven by improved availability and power of hardware, software and Wi-Fi, and also by growing class sizes [5]. Many educators have begun to use educational technology to allow learners to respond to and interact with materials, both within the face-to-face context and online [6, 7]. Enhancing involvement, participation, and maximum interactivity for both students and faculty is an area for potential improvement in medical education [8]. There is an increasing trend toward shifting from traditional teaching to student-centered teaching that actively engages students [9].

Creating interactivity within the classroom is becoming easier than ever with available educational technology tools [10]. One way of increasing interaction between educators and learners during a learning activity is via an audience response system (ARS). ARS is a relatively simple technology tool that allows educators to poll the audience and collect instant responses which can then be shared with all participants instantaneously [11]. Polling the audience instantly promotes further exploration and discussion of point of special importance. Furthermore, quizzing all students encourages individual engagement while also informing the instructor immediately of the students’ levels of understanding. Various methods have been used over the years [12]. However, rather than a "magic bullet" to educational woes, these systems are merely tools which can be used in a number of ways; therefore, defining what the ARS can add to a learning environment is required [13]. Examining the exact role in a particular learning environment provides further guidance of how to apply and better use such a tool. The ARS’ potential role and best practice in medical education was explored in this study.

Interactive education has been used to a limited extent in Iraq [14]. The use of educational technology is a relatively new experience to the medical education there [15]. The country has been through a prolonged period of political conflict and civil war. Only a very limited amount of educational technology has been introduced in a few Iraqi educational institutions [14]. These have been in the form of sporadic projects of limited application initiated by individuals or small groups and with little subsequent formal evaluation. However, any intervention must be acceptable to all stakeholders to have lasting utility [16]. Therefore, the aim of this study was to provide such a formal evaluation of using a simple introductory technology to enhance interactivity in large group teaching and improve our understanding about medical education in the context of the ARS in Iraq.

## Methods

The study was conducted in an Iraqi medical school (Wasit University) during the 2018-2019 academic year as part of introduction of new simple educational technology. The medical school follows the same national six-year curriculum (3 basic and 3 clinical years). The ARS has been considered for use as an introduction of a simple technology enhanced interactive education. Upon the completion of the course, the following queries were addressed: 1) students’ perception of the ARS use experience as assessed by a quantitative survey and a qualitative method using Focus Group Discussion (FGD) [17]; 2) and an evaluation of the instructor’s perception of the experience using a survey. Approval from the Dean’s office of Wasit University College of Medicine, the official licensing authority to approve all experiments in the university medical school, was obtained. There is no institutional or licensing committee at this university. Participation was voluntary and anonymized for the quantitative part (the survey questionnaire), and informed consent was obtained for the qualitative part (FGD).

ARSs are generally used in multiple ways: as a learning strategy to facilitate improving attention, interaction, instruction, student preparation and discussion, and formative and summative knowledge assessments. The particular use and setting of the ARS might determine the extent of its usefulness. It is therefore important to study and choose the appropriate setting before implementing ARS use, especially with changing educational styles and evolving technology.

The Head and Neck course was chosen due to the motivation and technology competency of the instructor who was willing to implement the new change. This course is taken in the third year of the curriculum. The course involved fifteen one-hour lectures over fifteen weeks. The ARS system with the associated software was installed and tested prior to the beginning of the course. This particular ARS system used a small, dedicated hand-held keypad to respond to questions posed by the educator with various different types of questions. At the beginning of the course, the instructor gave the students a short introduction in using ARS. He then used ARS-based questions throughout the lecture presentation. Three to five questions were incorporated in each lecture based on the instructor selection where deemed necessary to enhance interactivity. Questions were designed to evaluate understanding and enhance critical thinking.

Students’ participation in answering the lecturer’s questions using the ARS was voluntary; however, they were encouraged to participate to learn. At the end of the course, students were invited to participate anonymously in the study survey. Voluntary and anonymous participation were maintained in the survey. This was important for the validity of the study results as anonymity enhances the rate of response, accuracy of information, and validity [18]. There were no positive reward or gifts for participating in the study. Neither was there a punishment or negative reward for not participating in the study. After the survey, focus group discussion (FGD) was conducted in a sequential explanatory design to expand and strengthen the study conclusions [19–21].

### Survey questionnaire

**Fig. 1:**
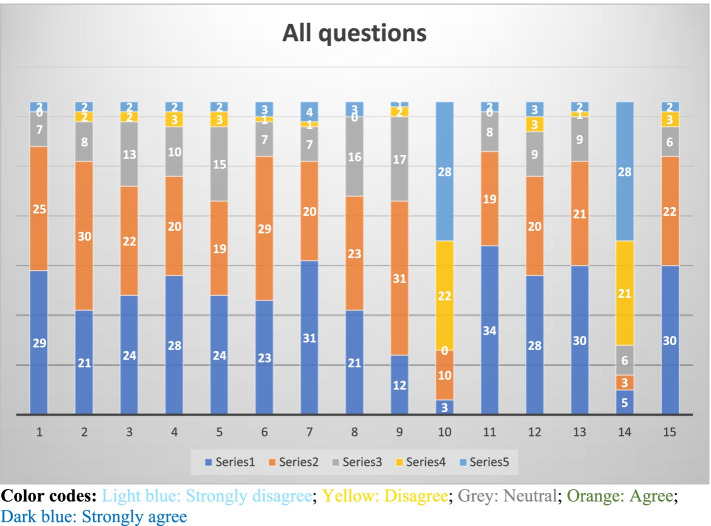
All questions from students’ survey (1-15) responses

A comprehensive survey was designed and structured for this study using the principles of evaluation. The main four dimensions of the evaluation of the educational activities (structure, process, instructor, and outcome) as discussed by Schiekirka et al. were considered. [22] Therefore, the survey questions covered the essential components of the educational activity, measuring the intended outcomes of students learning, focusing on the adult style of learning that uses learner-guided activities and goals for learning, and using as valid and reliable questions as possible. The survey questions were used with a small group as a pilot test before the main study. Modifications of some of the questions were accordingly applied. A 5-point Likert scale from “Strongly Disagree” to Strongly Agree” was used to measure responses to each question. Percentage calculations and interpretation of the answers on the fifteen survey questions (Table 1 and Fig. 1) were collected and described as both the mean and mode. The mode was used to define the most common response while the mean was employed to as a measure of central tendency [23].

**Table 1:** All answers by students’ survey questions with mean and mode

		**SD**	**D**	**N**	**A**	**SA**	**mean**	**mode**
#	Number assigned for calculating mean	1	2	3	4	5		
1 ^b^	The ARS helped to improve my attention and focus during the lecture	2 3%	0 0%	7 11%	25 40%	29 46%	4.25	SA
2 ^e^	The ARS helped me to understand the topics of the lecture better	2 3%	2 3%	8 13%	30 48%	21 33%	4.05	A
3 ^d^	The ARS stimulated me to discuss the topic with my colleagues and teacher	2 3%	2 3%	13 21%	22 35%	24 38%	4.02	SA
4 ^d^	The ARS stimulated me to prepare for the topic in advance	2 3%	3 5%	10 16%	20 32%	28 44%	4.10	SA
5 ^d^	The ARS stimulated me to study and review the topic more after the lecture	2 3%	3 5%	15 24%	19 30%	24 38%	3.95	SA
6 ^b^	The ARS helped me to memorize information more	3 3%	1 2%	7 11%	29 46%	23 36%	4.08	A
7 ^b^	The ARS motivated me to attend lectures	4 6%	1 2%	7 11%	20 32%	31 49%	4.16	SA
8 ^b^	The ARS helps me answer questions and participate with no embarrassment	3 5%	0 0%	16 25%	23 37%	21 33%	3.94	A
9 ^c^	Using ARS provided answers to some of the questions that I have about the topic	1 2%	2 3%	17 27%	31 49%	12 19%	3.81	A
^a^ 10 ^e^	Using ARS was a waste of time^a^	28 44%	22 35%	0 0%	10 16%	3 5%	3.98	SD
11 ^c^	I wish ARS used in all other subjects	2 3%	0 0%	8 13%	19 30%	34 54%	4.32	SA
12 ^e^	Using ARS made me like the topic more than other topics	3 5%	3 5%	9 14%	20 32%	28 44%	4.06	SA
13 ^c^	Using ARS encouraged me to use technology in learning	2 3%	1 2%	9 14%	21 33%	30 47%	4.21	SA
^a^14 ^c^	It was difficult to use the ARS^a^	28 44%	21 33%	6 10%	3 5%	5 8%	4.02	SD
15 ^c^	Overall, I find the ARS is an efficient tool of teaching	2 3%	3 5%	6 10%	22 35%	30 48%	4.19	SA
	**Total**	**38**	**34**	**138**	**344**	**391**	**4.08**	

^a^Q 10 & 14 were reverse scored for the mean

^b^Answers by attendance, engagement and memory domain

^c^Answers by learning preferences and use of technology domain

^d^Answers by adult and active learning domain

^e^Answers by learning efficiency/quality and the effect of educational technology domain

### Focus Group Discussion (FGD)

FGD aims to gain data about specific study parts from purposely selected participants. Participants were selected and a comfortable environment for the FGD was prepared. Open discussion was facilitated by an experienced moderator (FT) and systematic theme content analysis was subsequently performed. Recurrent thematic statements that were related to the educational experience and perception were used. The mixed method was used to verify and validate findings from the quantitative and qualitative data. This involved separate analysis of the two data sets combining and comparing the results. In this convergent use, the two types of data can create a stronger foundation for conclusions and validation of the results [24].

### Educator’s perception of using the ARS

As part of the educational process and experience, the educator’s perception is of great importance in estimating how ARS could become part of their educational curriculum in the future. A survey was therefore developed to assess the educational value and technical aspects from the instructor’s perspective. The survey was conducted at the end of the course and was divided into the preparation and lecture delivery phases. Similar to the student questionnaire, a 5-point Likert scale from “Strongly Disagree” to “Strongly Agree” was used to measure responses to each question (Appendix B).

### Study validity and reliability

Validity and reliability were considered and enforced at different levels of the study. Content validity that indicates the adequacy and effectiveness of measuring a variable was optimized by changing and adjusting some of the survey questions according to the pilot study. Internal validity was enhanced by using different ways of collecting data (survey and FGD). External validity in this study is challenging. The applicability of this study in other setting is difficult to determine since it is a single-site single-course study. However, this time-limited study will be considered a first step to promote further studies in related or different contexts before implementing the results.

External reliability that measures the replicability of the results is one of the known weaknesses of mixed method research. The different modalities of the data collection instrument and the availability of the participants are difficult to replicate. The internal reliability that reflects the consistency of data collection, analysis and interpretation has one weakness that is related to the analyzing of the FGD. This is an inherent interviewer variability with FGD. However, inter-rater, or inter-observer reliability was high considering the educational level and the scope of the authors’ practice.

## Results

### Survey

Sixty-three responses from all the participants in the course were obtained for all the fifteen questions (100% participation rate) (Table 1). The results strongly indicate that the students perceived several advantages of using the ARS. Within each domain, they demonstrate:

#### Attendance, engagement and memory

The majority of students agreed the ARS helped to improve attention and focus during lectures. Eight three per cent of students agree or strongly agree that the ARS helped with memorization of information, and 70% felt it helped answer questions without embarrassment. Most students (81%) strongly agreed the use of the ARS motivated them to attend lectures (Table 1).

#### Learning preferences and use of technology

There is predominant agreement on the overall usefulness of the ARS and their wish to use it in other courses. Sixty-eight percent (68%) of students with questions about a lecture topic had their questions answered using the ARS. Students strongly agreed that the use of the ARS encouraged the use of technology in their education (80%) and it was not difficult to use the ARS (77%). Participants (84%) wished to use this ARS in other courses (Table 1).

#### Adult and active learning

Most of the students (76%) felt that the ARS motivated them to prepare for lectures in advance. Sixty-eight per cent of the students agree or strongly agreed that the ARS stimulated them to study and review further after lecture, and 73% felt that it encouraged discussion with colleagues and instructors (Table 1).

#### Learning efficiency, quality, and technology effect

The majority of the students liked the ARS use and felt it improved their understanding (Table 1). Eighty-one percent (81%) of students agreed or strongly agreed the ARS augmented their learning, helping them to understand the topics of the lecture better. Most students (79%) disagreed or strongly disagreed that the ARS was a waste of time. Students also report they liked the topic more than other topics they have covered because of the use of the ARS (Table 1).

### Focus Group Discussion (FGD)

In reviewing the FGD content, the following themes emerged: disadvantages and advantages. Within each theme, a few subthemes became evident (Table 2). These are labelled Sn where n is consecutive number, to anonymize the respondents and to show a wide spread of contributors to the discussions.

**Table 2:** FGD Themes and frequencies

Caterory 1: Advantages				
Subcategory	Theme	Frequency	Students	Example Qoute
A	Summarizing the topic	2	S2, S4	“Summary of topics is good”
B	Improving focus	5	S2, S3, S4, S7, S8	“I like it and I can pay attention”
C	Thinking simulation	6	S1, S4, S5, S6, S7, S8	“we understand and ask questions”.
D	Helps preparing for the exam	1	S4	“It helps study for the exam”
E	Special way of learning.	2	S6, S7	“It is different from other subjects; I learn more here”
F	Entertaining	6	S2, S3, S4, S5, S6, S8	“We like it and it’s like playing game”
Total		22		
Caterory 2: Disadvantages				
Subcategory	Theme	Frequency	Students	Example Qoute
A	Time consuming	3	S1, S3, S8	We spend a lot of time and we wait for the answers
B	Repetition	1	S5	Every time it is the same things we answer questions and wait until we see the answers
Total		4		

#### I- Advantages:

A- *Use to summarize a topic.* Students felt the use of ARS to summarize topics was useful - “summary of topics is good’ (S2).
B- *Improving focus.* Several students commented on how the ARS focused their attention - ‘I like it and I can pay attention’ (S4).
C- *Thinking stimulation.* Several students also expressed better understanding and critical thinking with using the ARS - “we understand and ask questions” (S8).
D- *Helps preparing for the exam.* Students felt that the use of ARS helped them to prepare for the exam - “It helps study for the exam” (S4).
E- *Special way of learning.* Some students found learning with the ARS is a special way of learning - “It is different from other subjects; I learn more here” (S6).
F- *Entertaining.* Several students felt it was entertaining to learn with the ARS use - “We like it and it’s like playing game” (S8).

II- Students’ perceived disadvantages of the ARS use by subthemes with quotes examples are presented below:

1. *Time consuming.* Students expressed concerns about consuming lecture time on the ARS use - “We spend a lot of time and we wait for the answers” (S1)
2. *Repetition.* Some students felt that there is excessive repetition of steps in the ARS use - “Every time it is the same thing, we answer questions and wait until we see the answers” (S5).

The frequencies associated with categories and subcategories were used to adjudicate the weight of different responses [25, 26].

### Educator’s perception of ARS

The answers and results obtained from the educator’s survey are summarized in Table 3. The educator strongly agreed that the level of difficulty in learning and using the ARS was within his skill level. The educator felt the time consumed in preparing ARS questions for each lecture was reasonable and that it is practical and convenient for lecture preparation. He also agreed that the use of the ARS did not affect the amount of lecture content delivered per session. He strongly agreed there was more interaction, deeper thinking, and broader involvement from students while using the ARS.

**Table 3:** Educator’s perception of the ARS use

A	Preparation phase:	
1	Time consumed in learning how to use the ARS is reasonable.	Disagree
2	Time consumed in preparing ARS questions and slides for the lecture is reasonable.	Agree.
3	Level of difficulty in learning and using ARS is within my skills level.	Strongly agree.
4	Using ARS is practical and convenient in terms of lecture preparation.	Agree.
5	Preparing and incorporating ARS questions for each lecture requires reasonable efforts.	Neutral
B	Lecture delivery phase:	
1	Using ARS does not affect the amount of lecture content I can deliver.	Agree
2	I observe more interaction from students using ARS in the lecture.	Strongly agree.
3	I noticed more thinking and deeper involvement from students using ARS.	Strongly agree.
4	I feel more enthusiastic to teach more and better using ARS.	Strongly agree
5	I enjoy using ARS.	Strongly agree.
6	I feel I deliver a better-quality lecture using ARS.	Strongly agree.
7	I have a better sense of students understanding of the topic using ARS.	Strongly agree
8	ARS helps me tailor the lecture according to the students’ understanding and needs.	Agree
9	ARS helps me to evaluate students’ overall knowledge and performance.	Agree
10	Overall, I find the ARS is an efficient tool of teaching.	Strongly agree

The educator felt more enthusiastic about teaching with better-quality lectures while using the ARS. The ARS helped to tailor the lecture according to the students’ understanding and needs, while also helping to evaluate students’ knowledge and performance. Overall, the educator enjoyed using the ARS and felt that the ARS is an efficient tool for teaching.

## Discussion

The use of ARS in medical education is a new experience for many medical schools, including those in Iraq. The introduction of the ARS use has promoted the use of technology in education and enhanced the concept of interactive learning. Although there is an abundancy of studies evaluating the use of ARS in Western culture, it is important that contextualized studies are done. It is imperative to understand both student and tutor perceptions, and the impact on using such a technology, both from the view of the dynamics of the group and from student results. This study provides an early evaluation of this novel experience.

### Students’ perception

The results of the survey collectively demonstrate a positive preference and advantages of ARS use in all the four domains. ARS use was perceived as liked, preferred, wanted, entertained, and advantageous. Furthermore, students’ responses revealed that the ARS helped them to better understand the topic and stimulated them to further discussion and thinking. FGD analysis revealed that students liked the ARS mostly because it stimulated them to think more deeply. Questions in education are often used by teachers to stimulate building knowledge and critical thinking skills [27, 28]. When the questions are embedded within a lecture, this becomes an invitation to think more deeply about the content of the lecture. This is a first step of the thinking process followed by additional stimulation from the shared responses of the audience and comparing answers. Such answer comparisons are a natural form of learning [29, 30]. Students like to compare themselves and their responses to the classroom responses. This generates a second round of thinking, reviewing and comparing, especially when the responses are diverse. Depending on the setting of the group activity, this could be led by the course instructor for further interactive discussion and shared learning. Question 5 in the students’ survey, “The ARS stimulated me to study and review the topic more after the lecture”, addresses this point specifically. Two thirds of the students agreed or strongly agreed on this effect. This reflects the extended positive effect of the ARS use on continuing learning beyond the session. Motivation increases persistence in achieving learning goals [31].

The advantages in the domains of attendance, engagement, and memory were evident. Survey results showed perception of improving focus and attracting to attend the educational activity (lecture). Most students found the ARS helpful in participating and interacting in the lecture without embarrassment. Nelson [32] reported five out of six studies he reviewed favored learner interactions in ARS lectures. Facilitating sharing input helps increase the interaction. The students also found the system stimulating to prepare for the lecture and discuss the topic with their instructor and colleagues. Hassanin et al. reported that the ARS use encouraged students to discuss the topic with peer, in addition to improving engagement and attendance [33]. Attention and interaction of learners may have long term consequences on memory [34]. Focus and attention are indicators of engagement in the educational activity which is an essential component of adult interactive learning.

The use of technology and learning preferences domain was addressed by both the survey and FGD. The potential issue with the use of new technology difficulties did not seem to be an obstacle in the use of the ARS. On the contrary, most of the students found it stimulating to use technology in learning. The use of technology by student continues to grow worldwide, with students reporting they use desktop computers, interactive whiteboards, smart phones, and tablets [35]. The positive perception and openness to use technology in education is an important factor in the introduction of more technology in education with expanding the use and application.

The use of ARS technology provides excitement to learners [36]. This can lead to the possibility that the other features of the ARS use were liked because of other factors e.g., entertainment rather than real positive perception of the features. The Technology Acceptance Model (TAM) provides some explanation of the technology use behavior and intention by associating it with the attitude toward technology and ease of use [37]. This uncertainty needs further exploration.

There is clear evidence from the study of the multiple advantages of the ARS use as perceived by the students. As a new electronic tool, the ARS was attractive to the students. Entertaining tools and activities may influence the entire activity to the positive side [38]. Another factor that might have contributed is the novelty of the experience to the students and the medical school. Students expressed excitement to use this technology as the first medical school in the country of Iraq. The course organizer and moderator expressed similar excitement.

The disadvantages of using the ARS reported from the FGD were much fewer than the advantages. The two main disadvantages that were reported are “time consumed” and “repetition” of the question posting process. The extra time needed for the ARS use and its questions is a known issue and disadvantage [39]. Interestingly, the instructor did not believe that using ARS affected the amount of lecture content that was delivered.

### Educator’s perception

The instructor’s perception was generally positive to all the questions of the survey except the two technical questions. Educators using technology often complain about the extra time needed to use technology. But, this extra time to learn the new technology can be evaluated against the length or term of the technology use as well as with the importance of the technology. Technology acceptance and use by teachers has been the focus of prior research, e.g. the Technology Acceptance Model (TAM) was developed to explain the influential factors and mechanisms of technology use, including in classrooms [40]. It is, therefore, important to consider all factors that influence the adoption of the technology considered for use.

The course had one instructor which limits the data available for evaluation. Alternative way of evaluating the perception was to conduct personal interview that may provide in-depth perception input. However, the course was expected to be delivered by more than one instructor but ended up completed by one instructor for reasons out of the study control.

The instructor was very motivated and enthusiastic which may not reflect the average medical educator’s attitude and availability. According to Sharma and Srivastava, teachers who are willing to adopt new tools are motivated in adopting new teaching approaches [41]. Teachers often resist using new technologies in their classrooms because of the challenges of the new experience [42]. Instructors of courses that are planning to introduce the use of ARS should be prepared to spend initial extra time to learn how to use the technology. With the current level of technology use in all aspect of life and the wide exposure of people to it, learning ARS technology is not lengthy nor difficult. Easy students’ learning and adjustment to technology and the use of the ARS was observed in other studies [39, 43].

### Study limitations

The study evaluated one instructor’s perception through a written survey. More instructors’ perception, if available, would be more accurate. In addition, qualitative personal interview might provide more detailed and accurate input from the instructor.

Another limitation is the short-term use of the ARS and the lack of long term follow up. Long-term outcomes and knowledge retention were not embedded into this study due to time limitations but would be useful to study in the future. The scope of this study allows to focus on learning, understanding and short-term application of information. Larger and long-term studies can be designed to evaluate higher level of learning and knowledge retention.

## Conclusions and recommendations

This study showed a strong agreement on multiple perceived advantages of the ARS use in lectures. The ARS induced interactivity and improved the learning process during lectures. The instructor’s role is crucial in the introduction of such technology successfully. Educational technology will play an increasing and important part of the medical educational system. However, it is important to choose the appropriate technology for the specific educational purpose. This ARS study demonstrated the smooth implementation of technology with acceptable and manageable challenges.

Further studies and information are needed to select, prioritize, and design the optimal ARS use in the various educational environments. Caution should be exercised to avoid generalizations too quickly as there might be other factors that could determine the utility and advantages of using ARS in other types of courses and different educational activities. Ours was a relatively short course with immediate outcomes measurement that might be different than long term outcomes and for longer use.

## Supplementary Information

**Additional file 1.**

## Data Availability

The datasets generated and/or analyzed during the current study are available in the appendices.

## Abbreviations

ARS: Audience response system
FGS: Focus group discussions
TAM: Technology Acceptance Model
