# Supplementary material for: The educational value of an audience response system use in an Iraqi medical school
Source: BMC Med Educ. 2022 Apr 26;22:319. doi: 10.1186/s12909-022-03381-z (PMC9040241; doi:10.1186/s12909-022-03381-z)
Supplement: Supplementary file 1 — Additional file 1. [file 12909_2022_3381_MOESM1_ESM.docx]

# Appendices

## Appendix A: Students Survey of effect of ARS on Medical Students Learning in College of Medicine University of Wasit – Iraq

1- The ARS helped to improve my attention and focus during the lecture:

a-    Strongly Disagree

b-    Disagree

c-     Neutral

d-    Agree

e-    Strongly Agree

2- The ARS helped me to understand the topics of the lecture better:

a-    Strongly Disagree

b-    Disagree

c-     Neutral

d-    Agree

e-    Strongly Agree

3- The ARS stimulated me to discuss topics with my colleagues and teacher:

a-    Strongly Disagree

b-    Disagree

c-     Neutral

d-    Agree

e-    Strongly Agree

4- The ARS stimulated me prepare for the topic in advance:

a-    Strongly Disagree

b-    Disagree

c-     Neutral

d-    Agree

e-    Strongly Agree

5- The ARS stimulated me to study and review the topic more after the lecture:

a-    Strongly Disagree

b-    Disagree

c-     Neutral

d-    Agree

e-    Strongly Agree

6- The ARS helped me to memorize information more:

a-    Strongly Disagree

b-    Disagree

c-     Neutral

d-    Agree

e-    Strongly Agree

7- The ARS motivated me to attend lectures

a-    Strongly Disagree

b-    Disagree

c-     Neutral

d-    Agree

e-    Strongly Agree

8- The ARS helps me answer questions and participate with no embarrassment:

a-    Strongly Disagree

b-    Disagree

c-     Neutral

d-    Agree

e-    Strongly Agree

9- Using ARS provided answers to some of the questions that I have about the topic:

a-    Strongly Disagree

b-    Disagree

c-     Neutral

d-    Agree

e-    Strongly Agree

10- Using ARS was a waste of time:

a-    Strongly Disagree

b-    Disagree

c-     Neutral

d-    Agree

e-    Strongly Agree

11- I wish ARS used in all other subjects:

a-    Strongly Disagree

b-    Disagree

c-     Neutral

d-    Agree

e-    Strongly Agree

12- Using ARS made me like the topic more than other topics:

a-    Strongly Disagree

b-    Disagree

c-     Neutral

d-    Agree

e-    Strongly Agree

13- Using ARS encouraged me to use technology in learning:

a-    Strongly Disagree

b-    Disagree

c-     Neutral

d-    Agree

e-    Strongly Agree

14- It was difficult to use the ARS.:

a-    Strongly Disagree

b-    Disagree

c-     Neutral

d-    Agree

e-    Strongly Agree

15- Overall I find the ARS very useful:

a-    Strongly Disagree

b-    Disagree

c-     Neutral

d-    Agree

e-    Strongly Agree

## Appendix B: Educators Survey of effect of ARS on Medical Students Learning in College of Medicine University of Wasit – Iraq

1- Time consumed in learning how to use the ARS is reasonable:

a-    Strongly Disagree

b-    Disagree

c-     Neutral

d-    Agree

e-    Strongly Agree

2- Time consumed in preparing ARS questions and slides for the lecture is reasonable:

a-    Strongly Disagree

b-    Disagree

c-     Neutral

d-    Agree

e-    Strongly Agree

3- Level of difficulty in learning and using ARS is within my skills level

a-    Strongly Disagree

b-    Disagree

c-     Neutral

d-    Agree

e-    Strongly Agree

4- Using ARS is practical and convenient in terms of lecture preparation.

a-    Strongly Disagree

b-    Disagree

c-     Neutral

d-    Agree

e-    Strongly Agree

5- Preparing and incorporating ARS questions for each lecture requires reasonable efforts.

a-    Strongly Disagree

b-    Disagree

c-     Neutral

d-    Agree

e-    Strongly Agree

6- Using ARS does not affect the amount of lecture content I can deliver.

a-    Strongly Disagree

b-    Disagree

c-     Neutral

d-    Agree

e-    Strongly Agree

7- I observe more interaction from students using ARS in the lecture

a-    Strongly Disagree

b-    Disagree

c-     Neutral

d-    Agree

e-    Strongly Agree

8- I noticed more thinking and deeper involvement from students using ARS.

a-    Strongly Disagree

b-    Disagree

c-     Neutral

d-    Agree

e-    Strongly Agree

9- I feel more enthusiastic to teach more and better using ARS.

a-    Strongly Disagree

b-    Disagree

c-     Neutral

d-    Agree

e-    Strongly Agree

10- I enjoy using ARS

a-    Strongly Disagree

b-    Disagree

c-     Neutral

d-    Agree

e-    Strongly Agree

11- I feel I deliver a better-quality lecture using ARS

a-    Strongly Disagree

b-    Disagree

c-     Neutral

d-    Agree

e-    Strongly Agree

12- I have a better sense of students understanding of the topic using ARS.

a-    Strongly Disagree

b-    Disagree

c-     Neutral

d-    Agree

e-    Strongly Agree

13- ARS helps me tailor the lecture according to the students’ understanding and needs.

a-    Strongly Disagree

b-    Disagree

c-     Neutral

d-    Agree

e-    Strongly Agree

14- ARS helps me to evaluate students’ overall knowledge and performance.

a-    Strongly Disagree

b-    Disagree

c-     Neutral

d-    Agree

e-    Strongly Agree

15- Overall, I find the ARS is an efficient tool of teaching:

a-    Strongly Disagree

b-    Disagree

c-     Neutral

d-    Agree

e-    Strongly Agree
